# Galectin-4 expression is associated with reduced lymph node metastasis and modulation of Wnt/β-catenin signalling in pancreatic adenocarcinoma

**DOI:** 10.18632/oncotarget.2104

**Published:** 2014-06-13

**Authors:** Mina Maftouh, Ana I. Belo, Amir Avan, Niccola Funel, Godefridus J. Peters, Elisa Giovannetti, Irma van Die

**Affiliations:** ^1^ Department of Medical Oncology, VU University Medical Center, Amsterdam, The Netherlands; ^2^ Department of Molecular Cell Biology and Immunology, VU University Medical Center, Amsterdam, The Netherlands; ^3^ Biochemistry of Nutrition Research Center, and Department of New Sciences and Technology, Faculty of Medicine, Mashhad University of Medical Sciences, Mashhad, Iran; ^4^ Start-Up Unit, University of Pisa, Pisa, Italy

**Keywords:** Pancreatic ductal adenocarcinoma, Galectin-4, migration, lymph node ratio, primary PDAC cells, Wnt/β-catenin pathway

## Abstract

Galectin-4 (Gal-4) has been recently identified as a pivotal factor in the migratory capabilities of a set of defined pancreatic ductal adenocarcinoma (PDAC) cell lines using zebrafish as a model system. Here we evaluated the expression of Gal-4 in PDAC tissues selected according to their lymph node metastatic status (N0 vs. N1), and investigated the therapeutic potential of targeting the cross-link with the Wnt signaling pathway in primary PDAC cells.

Analysis of Gal-4 expression in PDACs showed high expression of Gal-4 in 80% of patients without lymph node metastasis, whereas 70% of patients with lymph node metastases had low Gal-4 expression. Accordingly, in primary PDAC cells high Gal-4 expression was negatively associated with migratory and invasive ability *in vitro* and *in vivo*. Knockdown of Gal-4 in primary PDAC cells with high Gal-4 expression resulted in significant increase of invasion (40%) and migration (50%, P<0.05), whereas enforced expression of Gal-4 in primary cells with low Gal-4 expression reduced the migratory and invasive behavior compared to the control cells. Gal-4 markedly reduces β-catenin levels in the cell, counteracting the function of Wnt signaling, as was assessed by down-regulation of *survivin* and *cyclin D1*. Furthermore, Gal-4 sensitizes PDAC cells to the Wnt inhibitor ICG-001, which interferes with the interaction between CREB binding protein (CBP) and β-catenin. Collectively, our data suggest that Gal-4 lowers the levels of cytoplasmic β-catenin, which may lead to lowered availability of nuclear β-catenin, and consequently diminished levels of nuclear CBP-β-catenin complex and reduced activation of the Wnt target genes. Our findings provide novel insights into the role of Gal-4 in PDAC migration and invasion, and support the analysis of Gal-4 for rational targeting of Wnt/β-catenin signaling in the treatment of PDAC.

## INTRODUCTION

Pancreatic ductal adenocarcinoma (PDAC) is the fourth most common cause of cancer-death, and has the worst prognosis of any major malignancy, with less than 5% of patients alive 5-years after diagnosis [1]. One of the major hallmarks of PDAC is its early systemic dissemination, which can be coupled to extensive local tumor spread [2]. These features explain why more than 80% of patients diagnosed with this disease cannot be offered surgical treatment and are among the main causes of the high mortality rate among PDAC patients.

Invasion and metastasis are complex processes, and further studies on their genetic and biochemical determinants are urgently needed. Loss of junctional contact between adjacent epithelial cells and cell-extracellular matrix association constitute fundamental prerequisites for cancer cell detachment from the primary tumor. However, not only adhesion molecules on the cell surface but also regulators of transmembrane signaling play a determinant role in tumor cell migration and regulate the potential for epithelial cells to metastasize [3].

Galectins consist of a large family of galactoside-binding soluble lectins that have been classified, based on their structure and carbohydrate-recognition domains, in prototype galectins (galectins-1, -2, -5, -7, -10, -11, -13, -14), chimera type (galectin-3), and tandem repeat type (galectins-4, -6, -8, -9, -12) [4]. Galectins display a broad variety of functions, including mediation of cell–cell interactions, cell–matrix adhesion and transmembrane signaling [5, 6]. The expression of galectins is strictly regulated during the development of individual cells, but can be altered under different pathological conditions [7]. In particular, galectins contribute to different key events in tumor cells, such as differentiation, angiogenesis, malignant transformation, apoptosis, tumor immune escape and resistance to anticancer drugs [8]. Galectins have also been described to modulate homo- and heterotypic adhesion of tumor cells thereby mediating invasion and metastasis in several tumor types, including PDAC [7, 9-11].

In this study we focus on the role of Galectin 4 (Gal-4) in PDAC. In healthy individuals Gal-4 is predominantly expressed in the luminal epithelia of the gastrointestinal tract, and not detected in healthy pancreas. Remarkably, in PDAC tissue a high expression of Gal-4 was observed both at the transcriptional and protein level [12, 13]. High expression levels of Gal-4 were shown in PDAC and in intraductal papillary mucinous carcinoma, while the almost invariably benign serous cystic neoplasms and mucinous cystadenoma of the pancreas showed no Gal-4 expression [12, 13]. These results lead the authors to consider Gal-4 to be a typical example of a gradual increase in transcriptional activity and to be a factor that could account for the invasive ability of PDAC.

Our recent studies, however, demonstrated an opposite role for Gal-4 showing that it was much higher expressed in PDAC cell lines with reduced migrating properties, compared to cells having metastatic abilities [14]. Moreover, Gal-4 delayed migration of PDAC cells both *in vitro* and *in vivo*, using the zebrafish experimental model [14].

In the current research we have extended these studies by exploring the role of Gal-4 in human PDAC tissues and in several primary PDAC cultures. Our data establish a role of Gal-4 as tumor suppressor in PDAC, since we showed that elevated Gal-4 levels correlated significantly with a reduced *in vitro* migratory and invasive behavior in primary PDAC cultures, as well as with a reduced lymph node metastasis in PDAC patients.

Moreover, we hypothesized that Gal-4 might inhibit metastasis by down-regulation of Wnt signaling target genes, as already shown for colon rectal cancer [15]. Recently, it was demonstrated that the activation of the Wnt/β-catenin pathway, which plays an essential role in proliferation and differentiation of many organs [16], is required for progression of PDAC [17]. In the absence of Wnt stimuli, GSK3-β phosphorylates β-catenin in order to degrade it. However, activation of this pathway results in dephosphorylation of β-catenin, followed by accumulation and translocation into the nucleus. Interaction of accumulated β-catenin with CREB binding protein (CBP) leads to an active transcriptional complex for downstream target genes [18], and appears a key step to activate transcription of target genes involved in PDAC development [17]. Enhanced Wnt/β-catenin signaling has been observed in human PDAC tissues and preclinical models, while inhibition of Wnt signaling through transfection with the Wnt inhibitors Icat and dn-Lef-1, or knockdown of β-catenin, increased apoptosis and decreased PDAC cells proliferation [19].

Thus, inhibition of Wnt/β-catenin signaling by novel anticancer agents might have a therapeutic impact on suppression of PDACs driven by this pathway, and key factors to identify these tumors are warranted. To this end, we here explored the interaction of Gal-4 with the Wnt/β-catenin signaling and demonstrated that Gal-4 sensitized PDAC cells to the Wnt inhibitor ICG-001, which disrupts the interaction between CBP and β-catenin.

## RESULTS

### Gal-4 expression in PDAC patients is associated with lack of tumor invasion in the lymph nodes

**Figure 1:**
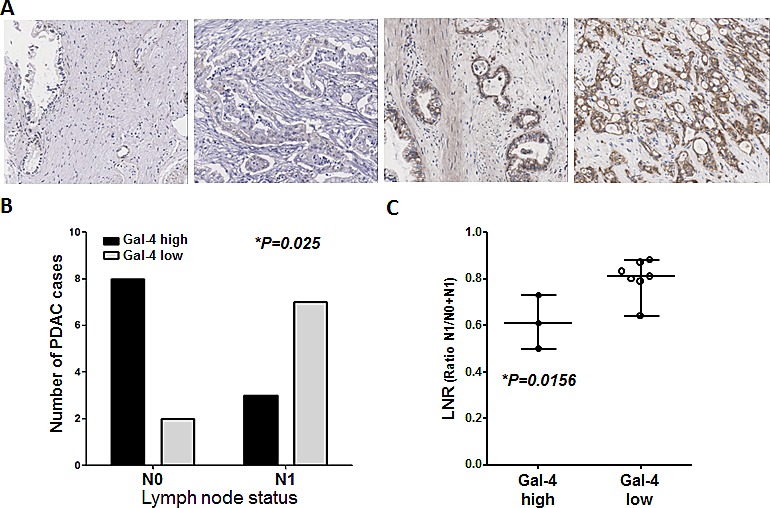
Patients with PDACs that highly express Gal-4 have a significantly decreased number of malignant cells in the lymph nodes, compared to patients with low Gal-4-PDACs (A) Representative pictures of immunohistochemical analysis for Gal-4 expression in PDAC patients, showing differential Gal-4 expression (negative, weak, intermediate, strong). (B) Patients were classified in two groups, i.e. with (N1) or without (N0) lymph node metastasis. IHC analysis showed that eight patients without lymph node metastasis had high Gal-4 expression, while two patients had low Gal-4 expression, whereas in the group of patients with lymph node metastasis three patients had high Gal-4 expression while seven patients had low Gal-expression. (C) Analysis of the LNR ratio in the group with lymph node metastasis (N1).

To explore the role of Gal-4 in PDAC invasive behavior we evaluated its expression in 20 PDAC patients selected according to their differential lymph node metastatic status. Gal-4 expression was heterogeneous and was detected both in PDACs and Pancreatic Intraepithelial Neoplasia (PanIN) lesions, while we did not observe stroma/background staining (Suplementary Fig. S1). As exemplified by the IHC pictures in the Figure 1A, some PDACs showed a negative or very weak staining, with a few positive cells, while other tumors had a higher number of positive cells, characterized by much stronger staining intensity. In order to take into account the potential heterogeneous staining of the tumors, we performed an analysis of all the pathological slides. Patients were categorized into two subgroups (low vs. high Gal-4 expression) with respect to the median protein expression (4 a.u.).

There was no difference in Gal-4 expression levels according to grade (P=*0.617*, two-sided Fisher's exact test), as well as for age, sex, neural infiltration, resection margins and occurrence of PanIN lesions. However, a trend toward a significantly higher percentage of tumors with vascular infiltration (P=0.092) was observed in patients' tumors with lower expression levels of Gal-4 (Table 1).

**Table 1:** Clinicopathological characteristics and association with Gal-4 expression

Characteristics		Gal-4 expression		
		Low (%)	High (%)	P*
Age, years	≤65	2 (22)	4 (36)	0.642
	>65	7 (78)	7 (64)	
Sex	Male	5 (56)	4 (36)	0.653
	Female	4 (44)	7 (64)	
Resection status	R0	6 (67)	9 (82)	0.617
	R1	3 (33)	2 (18)	
Vascular infiltration	No	7 (78)	4 (36)	0.092
	Yes	2 (22)	7 (64)	
Neural infiltration	No	1 (11)	2 (18)	0.999
	Yes	8 (89)	9 (82)	
Stage	T3N0Mx	2 (22)	8 (73)	0.070
	T3N1Mx	7 (78)	3 (27)	
Grading (WHO)	1-2	6 (67)	9 (82)	0.617
	3	3 (33)	2 (18)	
PanINs	No	5 (56)	8 (73)	0.642
	Yes	4 (44)	3 (27)	
Tumor size	< 20 mm	0 (0)	0 (0)	*n.d*.
	> 20 mm	9 (100)	11 (100)	

n.d., not determined

PanIN, Pancreatic Intraepithelial Neoplasia

WHO, World Health Organization

* P calculated with the two-sided Fisher's exact test

Furthermore, 80% of the patients without lymph node metastasis (N0) had high Gal-4 expression, while only two patients of this group had low Gal-4 expression. Conversely, considering the group of patients with lymph node metastasis (N1), most patients (70%) had a low level of Gal-4 expression (P=0.025; Figure 1B). Recent studies showed that not the lymph node involvement per se but especially the lymph nodes ratio is an independent, and one of the strongest prognostic factors after resection of pancreatic cancer [20]. Therefore, we performed also the analysis of the lymph node ratio (LNR), calculated as the ratio between the number of lymph nodes with metastasis and the number of examined lymph nodes. Remarkably, patients with low Gal-4 expression had a significantly higher LNR than patients with high Gal-4 expression (Figure 1C).

No significant correlations were observed for disease-free survival (DFS), but patients with Gal-4 expression below median had a trend towards significantly shorter overall survival (OS)(12.0 months, 95% Confidence Intervals (CI), 6.4-17.6, vs. 17.5 months, 95%CI, 15.0-20.1, P=0.072), as illustrated in the Supplementary Fig. 2. Similar results were observed for lymph node status (16.5 months, 95%CI, 5.1-27.8, in N0 vs. 13.4 months, 95%CI, 5.4-21.4, in N1 patients, P=0.077). Finally, among the 10 N1 patients (median OS, 13.6 months), 5 out of 5 of the patients with LNR>median (i.e. above 0.79) survived less than 14 months, whereas 4 out of the 5 patients with LNR≤0.79 survived more than 14 months. However, the small number of cases limited further statistical evaluation.

### Expression of Gal-4 in PDAC cells and xenografts

**Figure 2:**
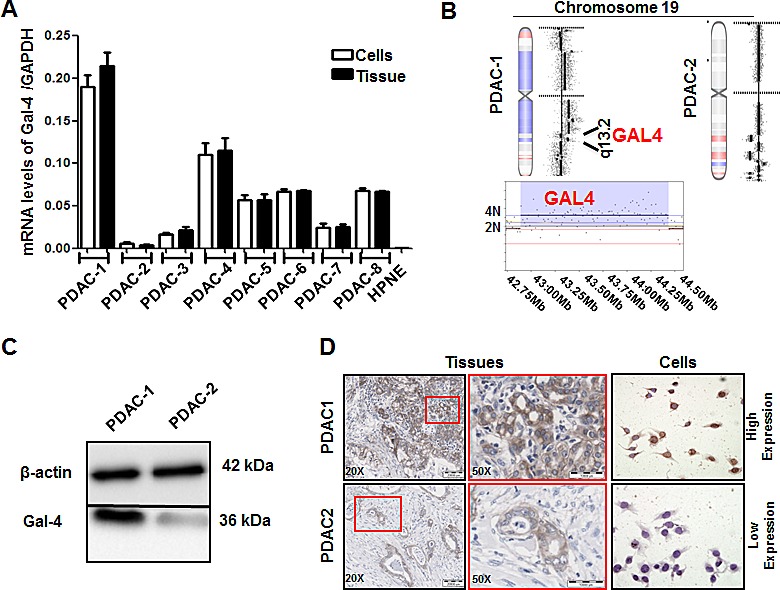
Gal-4 is differentially expressed in primary PDAC cell cultures, as well as in their originator tissues (A) Gal-4 mRNA levels in primary tumor cultures (white bars), and their originator tissues (black bars), as determined by qRT-PCR. Columns and bars represent the arithmetic means ± SEM of two independent experiments performed in triplicate. (B) aCGH analysis of copy number variations in the Gal-4 gene within the cytoband 19q13.2 of PDAC-1 and PDAC-2 cells shows a copy number gain (4N) in PDAC-1 cells. Left shifts and red color indicate the deleted segments, while right shifts and blue color indicate the gains/amplifications. The complete aCGH database is available at Gene Expression Omnibus (GEO) with accession number GSE44587. (C) Representative blots of Gal-4 protein expression in the PDAC-1 and PDAC-2 cells. As a loading control β-actin levels are indicated. (D) Representative pictures of Gal-4 protein expression in the PDAC-1 and PDAC-2 originator tissues and primary cells, with insets at higher magnification to allow evaluation of the intracellular pattern. Original magnification, 40X.

*Gal-4* was expressed in all the primary PDAC cell cultures tested, as well as in their originator tissues. However, this expression differed among cells, ranging from 0.006 a.u. in PDAC-2 cells, to 0.190 a.u. in PDAC-1 cells (Figure 2A). The mean (0.059±0.10 a.u.) and median (0.058 a.u.) expression levels in the tumor cells were significantly higher than the expression measured in the immortalized normal ductal cells hTERT-HPNE (0.002 a.u., P<0.01). Remarkably, *Gal-4* gene expression in the 8 primary tumor cells and their originator tumors showed a similar pattern and resulted highly correlated with Spearman analysis (R^2^>0.96, P<0.01), suggesting that these cells represent optimal preclinical models for studies on PDAC. PDAC-1 and PDAC-2 cells were selected for further studies, since they had the highest and lowest *Gal-4* expression, respectively. In these cells we explored copy number variations in the *Gal-4* gene, which are included in the cytoband 19q13.2, and we observed a copy number gain (4N) in PDAC-1 cells. Conversely, no changes were identified in PDAC-2 (Figure 2B). This data might at least in part explain the overexpression of *Gal-4* in PDAC-1 compared to PDAC-2 cells.

To investigate whether mRNA expression differences of Gal-4 observed in PDAC cells were reflected in differences in protein levels, we analyzed the protein expression of Gal-4 by Western blotting, ICC and IHC. These studies were performed in the PDAC cells and in their originator tumors, and demonstrated that PDAC-1 had markedly higher expression of Gal-4 protein with respect to PDAC-2 cells and tumors (Figure 2C-D). Furthermore, we confirmed the cytosolic staining pattern of Gal-4 in PDAC cells, as described previously [14].

### Gal-4 expression does not correlate with proliferation but correlates with invasive/migratory capabilities of PDAC cells

**Figure 3:**
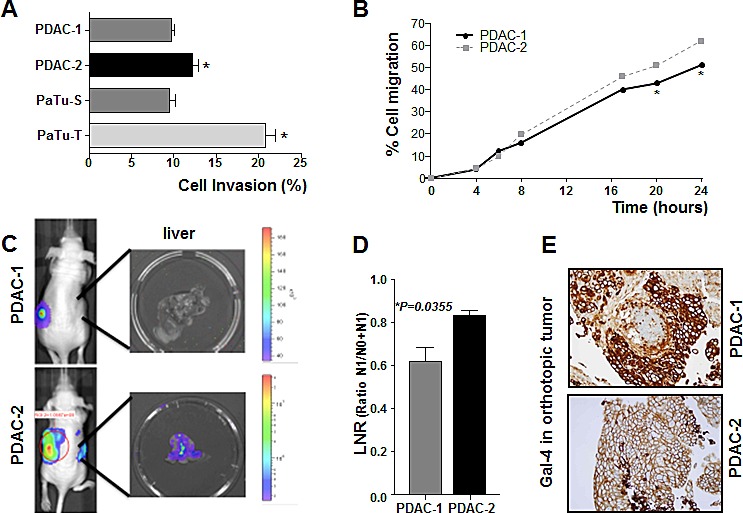
Gal-4 expression correlates with invasive and migratory capabilities of PDAC cells (A) Invasion of primary PDAC-1 and PDAC-2 cells characterized by high and low Gal-4 expression, respectively, as measured by migration over collagen-coated transwell chambers. Columns and bars represent the means ± SEM of two independent experiments performed in triplicate. *P<0.05 (B) Migratory properties of PDAC-1 and PDAC-2 cells as determined in wound-healing assay. Points and bars represent the means ± SEM of two independent experiments performed in triplicate. *P<0.05 (C) Representative Firefly-luciferase bioluminescence images of orthotopic mouse models, derived from PDAC-1 and PDAC-2, characterized by low and high metastatic properties, respectively. (D) LNR ratio in PDAC-1 and PDAC-2 orthotopic mouse models. Columns and bars represent the means ± SEM values in three mice for each group. (E) Representative IHC pictures of Gal-4 protein expression in PDAC-1 and PDAC-2 orthotopic mouse models. Original magnification, 40X.

We did not find any significant modulation of PDAC cell proliferation/survival according to differential Gal-4 expression, as reported in our previous studies [14]. However, our previous studies showed that low and high expression of Gal-4 in the PDAC cell lines PaTu-T and PaTu-S were associated with high and low migration and metastatic ability, respectively [14]. Here we further evaluated the invasive properties of these PDAC cells as well as the invasion and migration of two primary PDAC cell cultures, PDAC-1 and PDAC-2. Our data showed that PDAC-1 and PaTu-S cells, characterized by high Gal-4 expression, were less invasive compared to PDAC-2 and PaTu-T cells, showing low Gal-4 expression (Figure 3A). Similarly, the ability of PDAC-1 cells to migrate to the wound area after 24 hours was significantly (P<0.05) lower compared to PDAC-2 cells (Figure 3B).

Furthermore, we evaluated whether the differential invasive potential and expression levels of Gal-4 were retained in bioluminescent orthotopic mouse models which we recently developed using PDAC-1 and PDAC-2 cells [21]. Most of our orthotopic tumors metastasized to other organs such as lymph nodes, and liver, as detected via bioluminescence (Figure 3C), and then confirmed via light microscopy of three non-sequential serial sections stained with hematoxilin and eosin. In particular, macroscopic metastases were observed in all the livers of the PDAC-2 mice, while no liver metastases were detected in 33% of the mice of the PDAC-1 group. Moreover the LNR ratio in the PDAC-2 models was 1.4-fold higher than in the PDAC-1 models (Figure 3D).

These mouse models (3 mice for each group) showed key histopathological features of human PDAC in terms of tumor infiltration, PDAC-associated desmoplastic reaction, ductal characteristics and adenocarcinoma differentiation. All these tumors react positively to human specific antibodies directed against CK8/18, CK7, CK19, Ca19.9, EGFR, and CEA (data not shown). However, the PDAC-1 tumors showed a strong staining for Gal-4, while the PDAC-2 tumors had only a weak staining (Figure 3E), and thus recapitulated the immunophenotypes of the PDAC cells and originator tumors. Of note, PDAC-1 mice survived longer than PDAC-2 mice (i.e. 59 vs. 46 days), but the small number of animals did not warrant a statistically supported survival correlation, as reported previously [21].

### Modulation of Gal-4 expression alters the migratory and invasive behavior of PDAC cells

In order to gain further insights on the role of Gal-4 on the migratory and invasive behavior of PDAC, we used “gain- and loss-of-function models” by recombinant human Gal-4 lentiviral transduction to overexpress Gal-4, and Gal-4 siRNA treatment to reduce Gal-4 expression, respectively.

**Figure 4:**
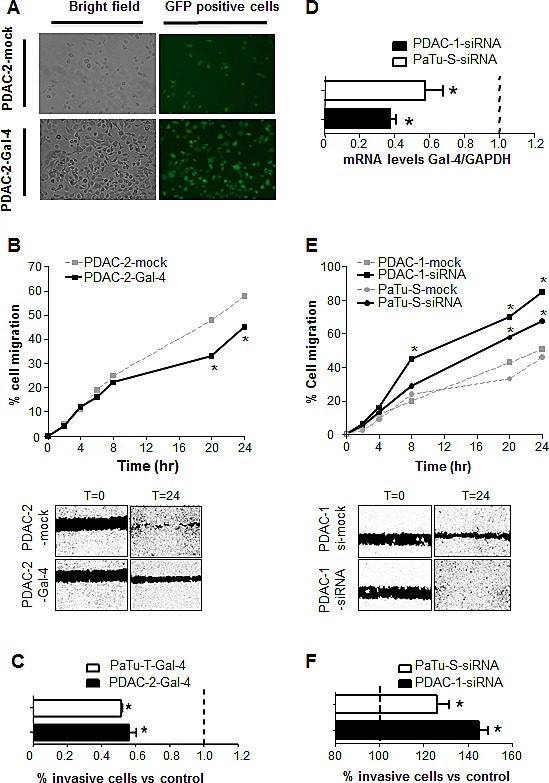
Modulation of Gal-4 expression alters the migratory and invasive behavior of PDAC cells (A) Representative pictures of fluorescence microscopy in PDAC-2-Gal-4-GFP cells. (B) Migratory properties of PDAC-2-Gal-4 cells, as determined in wound-healing assay. Points represent the means of three independent experiments performed in triplicate. *P<0.05. The photograph under the graph shows a representative picture at 24 hours. (C) Invasion of PDAC-2-Gal-4 and PaTu-T-Gal-4 cells, as measured by migration over collagen-coated transwell chambers. Data are expressed as percentage of invading cells compared to mock transduced PDAC-2 and PaTu-T cells, respectively. Columns and bars represent the means ± SEM of three independent experiments performed in triplicate. *P<0.05. (D) mRNA levels of Gal-4 in PDAC-1 and PaTu-S, both transfected with Gal4-siRNA, as determined by qRT-PCR. Columns and bars represent the means ± SEM of three independent experiments performed in triplicate. *P<0.05. (E) Migratory properties of PDAC-1 and PaTu-S cells both transfected with Gal-4 siRNA. Points represent the means of three independent experiments performed in triplicate. *P<0.05. The photograph under the graph shows a representative picture at 24 hours. (F) Invasion of PDAC-1-siRNA and PaTu-S-siRNA cells, as measured by migration over collagen-coated transwell chambers. Data are expressed as percentage of invading cells compared to mock treated PDAC-1 and PaTu-S cells, respectively. Columns and bars represent the means ± SEM of three independent experiments performed in triplicate. *P<0.05.

As shown in the Figure 4A, we successfully established Gal-4-overexpressing and mock-treated subclones, with more than 70% efficiency, in PDAC-2 cells. These subclones were tested for their differential migratory and invasion abilities using the wound healing and Boyden-chamber assays, as described above. The migration of PDAC-2-Gal-4 cells was significantly reduced compared to the PDAC-2-mock cells (i.e., -15% after 20 hours, Figure 4B). These Gal-4 overexpressing cells also had a 20% reduction (P<0.05) in the invasive behavior compared to the control cells (Figure 4C). Similar results were obtained with PaTu-T cells transduced with Gal-4 (Figure 4C).

To generate loss-of-function phenotypes we transduced PDAC-1 and PaTu-S cells with Gal-4 siRNA. As shown in Figure 4D, Gal-4 expression was significantly (P<0.05) decreased in both cell cultures, compared to the control, as determined by qRT-PCR. The Gal-4-siRNA transfection increased the invasive behavior of both PDAC-1 (+43%) and PaTu-S (+24%) cells (P<0.05, Figure 4E). Moreover, the knockdown of Gal-4 resulted in increased migratory ability, i.e. about 20% after 8 and 20 hours, in PDAC-1 and PaTu-S cells compared to their control cells (P<0.05, Figure 4F).

Conversely, modulation of Gal-4 in both our gain- and loss-of-function models did not modulate cellular proliferation, as described above in the primary cultures and in PDAC cell lines in our previous studies [14].

### Gal-4 reduces β-catenin levels and migration of PDAC cells through modulation of the Wnt pathway

**Figure 5:**
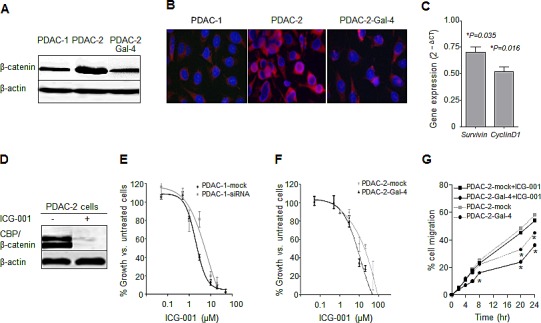
Gal-4 modulates β-catenin levels and sensitizes PDAC cells to the Wnt inhibitor ICG-001 (A) Representative pictures of β-catenin protein expression as detected by Western blot in PDAC-1, PDAC-2 and PDAC-2-Gal-4 cells. As a loading control β-actin levels are indicated. (B) Immunofluorescence analysis of the accumulation of β-catenin (red) into the nucleus (blue), in PDAC-1, PDAC-2 and PDAC-2-Gal-4 cells. (C) mRNA levels of *survivin* and *cyclin-D1* in PDAC-2-Gal-4 cells, as detected by quantitative RT-PCR. The results are calculated with the ΔCt method compared to mRNA levels of PDAC2 cells (set at 1). Columns and bars represent the means ± SEM of two independent experiments performed in triplicate. (D) β-catenin protein levels in PDAC-2 cells, untreated versus treated with ICG-001, as assessed by immunoprecipitation. (E) Inhibition of cell proliferation in PDAC-1-mock and PDAC-1-Gal4-siRNA after 72 hours exposure to the Wnt inhibitor ICG-001. Points and bars represent the means ± SEM of three independent experiments performed in triplicate. *P<0.05. (F) Inhibition of cell proliferation in PDAC-2-mock and PDAC-2-Gal-4 cells, after 72 hours exposure to the Wnt inhibitor ICG-001. Points and bars represent the means ± SEM of three independent experiments performed in triplicate. *P<0.05. (G) Migratory properties of PDAC-2-mock and PDAC-2-Gal-4 cells exposed to ICG-001 at IC50 concentration, as determined in wound-healing assay. Points represent the means of two independent experiments performed in triplicate. *P<0.05.

Since previous studies in colorectal cancer showed that Gal-4 can modulate β-catenin expression [15], we evaluated the expression of β-catenin in PDAC-1, PDAC-2 and PDAC-2-Gal-4 by Western blotting. As illustrated in Figure 5A, PDAC-2 cells presented a band with high density, while a fade band was observed in PDAC-1 cells. However, the transduction of the PDAC-2 cells with human Gal-4 significantly (P<0.05) reduced the expression of β-catenin (Figure 5A). PDAC-2 cells were characterized also by higher accumulation of β-catenin into the nucleus, whereas Gal-4 transduction reduced this accumulation, as demonstrated by immunofluorescence analysis (Figure 5B). Importantly, Gal-4 overexpression in PDAC-2 cells leaded to down-regulation of key target genes in the Wnt pathways, such as *survivin* and *cyclin D1* (Figure 5C). Keeping with these findings, we observed that cells overexpressing Gal-4 were arrested at G1/S (data not shown).

Finally, we evaluated the role of Gal-4 expression on cell proliferation as well as on sensitivity of the cells to the specific Wnt inhibitor ICG-001. Interestingly, our results show for the first time in PDAC-2 cells that ICG-001 disrupted β-catenin/CBP complex, as assessed by immunoprecipitation (Figure 5D).

**Figure 6:**
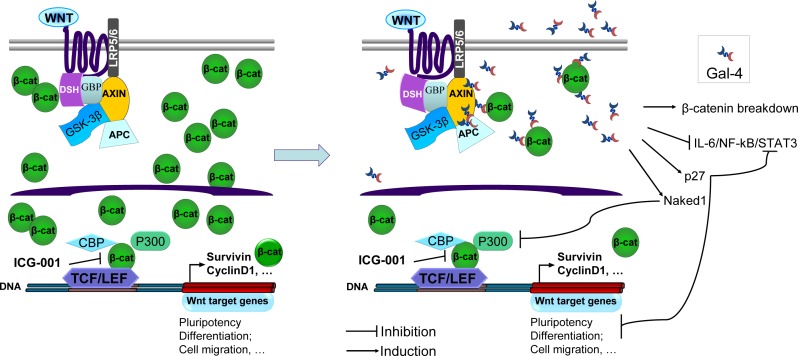
Model for Gal-4 effects on canonical Wnt signalling Upon activation of the canonical Wnt signaling pathway, Frizzled (dark blue line) and low-density lipoprotein receptor-related protein 5/6 (LRP5/6) activate the protein Dishevelled (DSH), leading to Axin recruitment. The β-catenin (β-cat) “destruction complex”, composed of the proteins Axin, adenomatous polyposis coli (APC), glycogen synthase kinase-3 (GSK-3β) and GSK3-binding protein (GBP), is not able to phosphorylate β-cat, resulting in its accumulation and translocation into the nucleus. Interaction of nuclear β-cat with CREB-binding protein (CBP) leads to an active transcriptional complex for downstream target genes by binding to T-cell factor (TCF)- and lymphoid enhancer-binding protein (LEF)-family transcription factors. The Wnt inhibitor small molecule ICG-001 specifically binds to CBP thereby disrupting the interaction of CBP with β-cat. In the presence of Gal-4, overall β-cat levels in the cell are decreased and thus less ICG-001 is required to disrupt the interaction of CBP with β-catenin. Gal-4 can bind to axin, β-cat and APC, as shown in CRC [15]. Possibly, Gal-4 cross-links these components, thereby stabilizing the destruction complex, and enhancing degradation of β-cat. Furthermore, Gal-4 is shown to inhibit the IL-6/NF-kB/STAT3 pathway [11], to induce expression of the Wnt signaling inhibitor protein Naked1, and is involved in maintaining p27 levels in CRC [15, 43, 44]. Collectively, these effects due to involvement of Gal-4 might contribute to reduced cell migration.

As shown in Figure 5E-F, ICG-001 inhibited PDAC-1 cell growth with IC50 values of 2.3 μM and 11.3 μM in PDAC-1 and PDAC-1-siRNA-Gal-4 cells, respectively. This compound was able to inhibit the cell proliferation with IC50 values of 22.8 μM and 0.3 μM in PDAC-2 and PDAC-2-Gal-4 cells, respectively. Thus, knockdown of Gal-4 in PDAC-1 cells increased IC50 values more than 5-fold, while enforced expression of Gal-4 in PDAC-2 cells decreased the IC50 about 76-fold. Similarly, ICG-001 was able to significantly reduce migration of PDAC-2-Gal-4 cells, compared to PDAC-2-mock cells, even after only 8-hour exposure (Figure 5G). This indicates that in the presence of Gal-4 less ICG-001 is required to disrupt the CBP-β-catenin complex and to inhibit migration. Collectively, these data indicate that Gal-4 acts by reducing cytoplasmic β-catenin levels, possibly by stabilizing the Axin-APC-GSK complex that promotes β-catenin degradation, resulting into lowered availability of nuclear β-catenin, and consequently diminished levels of nuclear CBP-β-catenin complex and reduced activation of the Wnt target genes (Figure 6).

## DISCUSSION

This is the first study demonstrating the role of galectin 4 (Gal-4) in the inhibition of the invasive/metastatic behavior of primary PDAC cell cultures, *in vitro* and *in vivo*, as well as in human samples. Remarkably, enforced expression of Gal-4 reduced cell migration and invasion. Furthermore, Gal-4 sensitized PDAC cells to the Wnt inhibitor ICG-001. As Gal-4 markedly reduced β-catenin levels, and was mostly observed in the cytoplasm, our data suggest that Gal-4 reduces cytoplasmic β-catenin levels, leading to the reduced transfer of β-catenin to the nucleus and subsequent interaction between CBP and β-catenin.

Recent gene array analyses of pancreatic cancer specimens, searching for genes that are linked to tumor progression, have raised interest in Gal-4. Gal-4 is a member of a large family of soluble carbohydrate-binding proteins that are well known to control tumor progression by promoting transformation, angiogenesis and immune escape [7, 14, 22]. In healthy individuals Gal-4 is predominantly expressed in the luminal epithelia of the gastrointestinal tract, where it acts as an innate immune mediator with bactericidal activity [23]. Remarkably, whereas Gal-4 is not found in healthy pancreas, it shows a significantly higher expression in cystic tumors of the human pancreas and in PDAC, compared to normal pancreas and benign neoplasms [12]. Similarly, Gal-4 expression is aberrantly induced in several tumors outside the pancreas, including breast and colorectal cancers [9, 15]. The latter data suggest that tumors may benefit from high expression of Gal-4. However, Gal-4 expression is strongly reduced in colon adenomas, and essentially undetectable in invasive colon carcinomas [15], indicating a function of Gal-4 as tumor repressor. Similarly, a genomic study showed a down-regulation of the expression of the *Gal-4* gene (LGALS4) in never-smoker lung tumors, suggesting the role of DNA methylation as a potential regulator of the expression of this gene [24].

We recently showed that Gal-4 expression inhibits the invasive behavior in two established PDAC cell lines, PaTu-S and PaTu-T, using *in vitro* migration assays and a zebrafish metastasis model [14]. Collectively, these data raise questions about the putative biological role of Gal-4 in PDAC, compared to its role in colon cancer and other tumor types.

Here we demonstrated for the first time that the LNR (ratio between metastatic and examined lymph nodes) was significantly lower in PDAC patients with high expression of Gal-4, compared to patients with low Gal-4 expression. Moreover PDAC patients with high expression of Gal-4 showed a trend toward a significant longer survival, suggesting that Gal-4 expression can serve as a prognostic factor in resected PDAC patients. Interestingly, Gal-4 expression was not correlated with grading or other clinicopathological features, suggesting its potential role as a novel marker for disease characterization. According to the final results of the CONKO-001 and ESPAC-3 trials, adjuvant chemotherapy increased disease-free survival and OS duration [25, 26]. Still, the identification of new prognostic factors for survival appears to be critical for the selection of patient subsets for better clinical management. The most biologically aggressive PDACs, such as those that recur soon after resection, should be treated initially with systemic therapy, as opposed to major surgery, which exposes the patient to substantial operative risk with little expected benefit. On the other hand, patients with indolent cancers may benefit from an aggressive surgical approach [27]. Moreover, prognostic biomarkers provide mechanistic insights into cancer progression, and might unravel molecular targets for novel treatment strategies [28].

High impact bench-to-bedside research on hundreds of patient samples improved prognostic capabilities in several tumor types, such as breast cancer [29]. Similar studies are difficult in PDAC, an organ with high endogenous nuclease activity, and a very small amount of tumor tissue available. A compendium of ~2500 published candidate biomarkers in PDAC was compiled [30], but several studies used tissues that were not microdissected to separate cancer from stroma, and 74% of the biomarkers in this compendium are based solely on mRNA evidence. Molecules that have both mRNA and protein evidence, such as Gal-4, are high-priority candidates for further testing. In particular, prospective studies in independent cohorts of patients will be crucial to validate our clinical findings on Gal-4.

In order to increase our understanding on the molecular mechanisms underlying PDAC biological aggressiveness as well as for rational planning of future therapeutic strategies, we performed comprehensive preclinical studies on Gal-4 activity in PDAC. Exploration of the expression and function of Gal-4 in eight primary PDAC cultures and their originator PDAC tissues indicated that the level of Gal-4 expression showed a similar pattern in the primary PDAC cultures and their originator tumors, establishing the value of the primary cell lines as a model to study PDAC. The tissue and resulting cell culture PDAC-1 was found to have the highest Gal-4 expression, which may be caused by amplification at 19q13.2. Conversely, the PDAC-2 tissue and cells showed the lowest expression levels of Gal-4. It is remarkable that the PDAC cells display a gain of copy number of a potential tumor suppressor gene. This suggests that PDAC cells may profit from expression of Gal-4, possibly in an early stage of tumor development. In the present study as well as in our previous research [14] we did not observe an effect of Gal-4 on proliferation/survival under normal growth conditions; however, it has been reported that upon expression of Gal-4, epithelial cells acquire the ability to survive lack of nutrients and growth factors for prolonged time [31]. This similarly may occur in PDAC, potentially contributing to tumor cell survival. In addition, PDAC cells may profit from expression of Gal-4 (which is secreted by the PDAC cells) by escaping from immune surveillance, for example by induction of T-cell apoptosis and expansion [32], or by promoting the development of anti-inflammatory cells such as tumor associated macrophages [33].

Importantly, we here show that Gal-4 expression inhibits the capacity for migration and invasion in primary PDAC cells. Furthermore, in the present study we demonstrated that tumors in a PDAC-1 mouse model showed a strong staining for Gal-4 and no liver metastases were detected in 33% of the mice, while the PDAC-2 tumors had only a weak staining and metastases were found in all livers. These results are in agreement with our previous data showing that cytosolic Gal-4 inhibits migration and metastasis in the PDAC cell lines PaTu-S and PaTu-T, using *in vitro* scratch assays, and an *in vivo Danio rerio* metastasis model, respectively [14].

Activation of Wnt/β-catenin pathway plays an important role in progression of PDAC [17]. Preclinical models throughout the last decade have established this pathway as an attractive drug target. However, therapies meant to attenuate the Wnt pathway have remained largely theoretical and preclinical [34], and key factors to identify tumors driven by this pathway are warranted. Therefore, in the present study we hypothesized that Gal-4 might inhibit metastasis by down-regulation of Wnt signaling target genes, as shown for colorectal cancer [15]. In keeping with this hypothesis, we here show evidence that Gal-4 interferes with Wnt/β-catenin signaling in primary PDAC cultures. The levels of β-catenin and its accumulation in the nucleus was significantly lowered in primary PDAC-1 cells that express higher protein levels of Gal-4. This is of significant relevance because the availability of β-catenin to translocate to the nucleus and activate downstream Wnt signaling target genes is required to initiate and critical for progression of PDAC [17].

In addition, Gal-4 expression increased the sensitivity to the specific Wnt inhibitor small molecule ICG-001. This small molecule is known to bind specifically to CBP disrupting the interaction of CBP with β-catenin. Treatment with ICG-001 induces apoptosis in colon carcinoma cells but not in normal colonic epithelial cells [35]. We showed an inverse correlation between Gal-4 expression and sensitivity of PDAC cells to the inhibitor ICG-001. In particular, IC50 values were 2 fold decreased when Gal-4 was forcedly expressed in PDAC-2, while suppressing Gal-4 expression in PDAC-1 cells increased the IC50 value more than 5 fold. This inverse correlation clearly shows that Gal-4 expression sensitizes tumor cells to this drug. Thus, inhibition of Wnt/β-catenin signaling by novel anticancer agents might have a therapeutic impact on suppression of PDACs driven by this pathway in Gal-4 high expressing pancreatic tumors.

In summary, our data establish a role of Gal-4 as tumor suppressor in PDAC, since we showed that elevated Gal-4 levels correlated significantly with reduced lymph node metastasis in PDAC patients, as well as with reduced *in vitro* migratory and invasive behavior in primary PDAC cultures, and reduced liver metastasis in mice. In addition our data support a role of Gal-4 in inhibition of the Wnt signaling pathway, thereby inhibiting migratory properties of the cells. Inhibition of Wnt/β-catenin signaling by novel anticancer agents might have therapeutic impact on suppression of PDACs driven by this pathway, and future translational and clinical studies on the role of Gal-4 in this process are warranted.

## MATERIALS AND METHODS

### Studies in clinical samples

#### Patients and immunohistochemistry (IHC)

In order to evaluate Gal-4 expression in human PDAC tissues, immunohistochemistry (IHC) was executed according to standard procedures in formalin-fixed paraffin embedded (FFPE) sections of 20 primary PDACs radically resected patients, carefully selected according to their clinicopathological characteristics. All these patients underwent radical surgical resection with curative intent (pancreatico-duodenectomy, total pancreatectomy and distal pancreatectomy) at the Department of General Surgery and Transplant, University Hospital of Pisa (Pisa, Italy), between 2000 and 2010 and were retrospectively reviewed using electronic medical records. Among them, we selected 10 patients with T3N0Mx stage, whereas the other 10 patients had N1 lymph node metastasis (i.e. T3N1Mx stage, according to the American Joint Committee on Cancer (7th ed. AJCC-2009) TNM staging system). The fact that all these patients underwent radical resection and pathological examination at one centre University Hospital of Pisa (Pisa, Italy), guaranteed the reliability of sampling, histological grading and immunohistochemistry analysis, as well as of evaluation of clinical outcome. Clinical data were available for all these patients.

Staining with the goat anti-human Gal-4 (1:100; R&D systems) was visualized with the BenchMark Special Stain Automation, and evaluated using a four-tier system, including positive cells number and intensity. In particular, the immunostaining intensity was classified into four grades: 0 (absent), 1 (weak), 2 (moderate), 3 (strong), as described previously [36]. We attributed one, two, or three additional points if the percentage of positive cells was less than 25%, 25% to 50%, or greater than 50%, respectively. Samples were defined as “high Gal-4”, when staining score was ≥median, and “low Gal-4” when staining score was<median (in arbitrary units, a.u.). Negative controls were obtained by replacement of primary antibody with buffer. All slides were reviewed by two researchers (N.F and E.G) who also evaluated the amount of tissue loss, background staining and overall interpretability before the formal Gal-4 reactivity evaluation. Additional digital analyses were performed with a computerized high-resolution acquisition system (D-Sight, Menarini, Florence, Italy), equipped with the automated quantitative image analysis software algorithm DSight software 2.1.0. The two groups of analyses were compared by t-test (P<0.05 for significant results).

### Preclinical studies

#### Chemicals and reagents

RPMI-1640 and DMEM medium, foetal bovine serum (FBS), penicillin (50 IU/mL) and streptomycin (50 μg/mL) were purchased from Gibco (Gaithersburg, MD). The Wnt inhibitor ICG001 was obtained from Selleckchem (Bio-Connect Diagnostics BV, Huissen, the Netherlands). Gal-4 siRNA and Silencer®Negative control were from Ambion (Life Technology, Bleiswijk, the Netherlands). Lipofectamine® LTX and Opti-MEM® were purchased from Invitrogen (Life Technology).

#### Cell culture

Eight primary PDAC cell cultures (PDAC1, PDAC-2, PDAC-3, PDAC-4, PDAC-5, PDAC-6, PDAC-7 and PDAC-8) were isolated from patients at the University Hospital of Pisa (Pisa, Italy), as described previously [21], while the human pancreatic duct epithelial-like cells hTERT-HPNE, and the PDAC cell lines Pa-Tu-8988T (PaTu-T) and Pa-Tu-8988S (PaTu-S) were purchased from the American Type Culture Collection (ATCC, Manassas, VA) and DSMZ-German Collection of Microorganisms and Cell Cultures (Braunschweig, Germany), respectively. The primary cells were cultured in RPMI-1640, supplemented with 10% heat-inactivated (HI) FBS and 1% streptomycin/penicillin at 37°C, in a 5% CO_2_ humidified atmosphere and harvested with trypsin-EDTA in their exponentially growing phase. The cell lines hTERT-HPNE, Pa-Tu-8988S (PaTu-S) and Pa-Tu-8988T (PaTu-T), PaTu-T cells lentiviral transduced with Gal-4 (PaTu-T/Gal-4) or lentiviral mock-transduced (PaTu-T/Mock) were cultured in DMEM high glucose (GIBCO, Invitrogen), with 10% HI-FBS and 1:100 streptomycin/penicillin.

#### Quantitative Real Time-PCR (qRT-PCR)

Total RNA was extracted using TRIzol® (Life Technology), yield and purity of the samples were checked at 260-280 nm with NanoDrop®-1000-Detector (NanoDrop-Technologies, Wilmington, NC). One μg of RNA was reverse transcribed using the DyNAmo cDNA Synthesis Kit (Thermo Scientific, Vantaa, Finland), according to the manufacturers' instruction. RNA isolation for the analysis of *Gal-4* expression in the primary cell cultures was performed within 10 passages. Moreover, in order to evaluate whether the expression of *Gal-4* was similar in the primary cells and the tumors from which they were derived, we also extracted RNA from these 8 tumors, after laser-microdissection with a Leica LMD7000 instrument (Leica, Wetzlar, Germany), using the QiaAmp RNA micro-Kit (Qiagen, San Diego, CA). Areas with morphological defined cancer cells were selected, and the precision of the laser beam resulted in the capture of individual cells with high degree of accuracy as described previously [37]. Quantitative Real Time-PCR (qRT-PCR) of *Gal-4* (*LGALS4*, NCBI Reference Sequence: NM_006149.3) was performed with the SYBR Green method in an ABI-7900HT sequence detection system (Applied Biosystems, Life Technology, Forster City, CA), as described previously [14]. Results were expressed as ratio of the threshold cycle (Ct) values and reported as arbitrary units (a.u.). Additional qRT-PCR studies evaluated the mRNA expression of *cyclin-D1* (*CCDN1*, NCBI Reference Sequence: NM_053056.2) and *survivin* (*BIRC5*, NCBI Reference Sequence: NM_001012271.1) using primers and probes from Applied Biosystems Assay-on-Demand Gene expression products (Hs00765553_m1 and Hs00153353_m1, respectively). The PCR was performed in a 25 μl reaction volume containing TaqMan Universal master mix (Applied Biosystems), in triplicate using the ABIPRISM-7500 sequence detection system instrument (Applied Biosystems), as described previously [38].

#### Array Comparative-Genomic-Hybridization (aCGH)

In order to evaluate whether different expression levels of Gal-4 were determined by copy number variation of this gene, genomic DNAs were extracted from PDAC-1 and PDAC-2 cells using Ambion®-RecoverAll kit (Life Technologies). The quantity and purity of extracted DNAs were determined as described above. DNAs were subjected to aCGH, using Agilent 4×180K platform (Agilent, Santa Clara, CA), as described previously [21]. The slides were scanned on Agilent Microarray Scanner, followed by data extraction and normalization by Feature Extraction v10.5 software (Agilent). Nexus Copy Number™ software was used to analyze the DNA copy number variations (BioDiscovery, Hawthorne, CA).

#### Western blot

Further analyses on Gal-4 and β-catenin protein expression were performed in PDAC-1, PDAC-2, Patu-S and Patu-T cells, which were selected for their differential *Gal-4* mRNA expression and migration capabilities as described earlier [14, 21].

Briefly, 30 μg of proteins was separated on a 10% SDS-polyacrylamide gel and transferred onto PVDF membrane (Immobilion®-FL, Millipore, Billerica, MA). The membrane was blocked with Rockland (Rockland inc., Pennsylvania, USA), and incubated with goat anti-human Galectin-4 (1:1000; R&D systems), mouse anti-β-catenin (1:1000; Cell Signaling, Danvers, MA) and mouse anti-β-actin (1:50000; Sigma–Aldrich Chemicals). The membrane was probed with the anti-mouse-InfraRedDye (1:10000, Westburg, The Netherlands) or anti-goat InfraRedDye (1:10000) secondary antibodies. Fluorescent proteins were determined by an Odyssey Infrared Imager (LI-COR Biosciences, Lincoln, NE), at 84-μm resolution, 0-mm offset, using high quality settings. The intensities of protein bands were quantified using the Odyssey v.3.0 software (LI-COR Bioscience), as described earlier [39].

#### Immunocytochemistry and IHC in cells and xenografts

For immunocytochemical studies (ICC) of Gal-4 expression, the cells were seeded in 8-well chamber slides (Lab-Tek-II Chamber Slide System, Nalge-Nunc, Naperville, IL). After 24 hours, the cells were fixed with 70% ethanol for 10 minutes. ICC was performed using goat anti-human Gal-4 (1:100), as described above for IHC. The cells were then stained with avidin-biotin-peroxidase complex (UltramarqueTM-HRP-Detection, Greenwood, AR), as described previously [39]. Negative controls were obtained replacing the primary antibody with PBS. The sections were reviewed and scored blindly by comparing the staining of PDAC-1 cells versus PDAC-2 and HPNE cells.

Additional IHC analyses of Gal-4 expression were performed in FFPE sections of PDAC specimens obtained from orthotopic mouse models of PDAC-1 and PDAC-2 cells, as described previously [39].

#### Overexpression and knock down of Gal-4 in PDAC cells

In order to investigate the effects of the modulation of Gal-4 expression on cellular processes, a specific human Gal-4 construct was generated and transduced into PDAC-2 and PaTu-T cells, as described previously [14]. Briefly the human Gal-4 (hGal-4) gene was cloned by inserting entire hGal-4 open reading frame in the vector pRRL-cPPT-CMV-X2-PRE-SIN-IRES-eGFP under a constitutive active CMV promoter. Lenti-viral productions containing the Gal-4 insert and the empty vector as well as subsequent infection of the primary PDAC-2 cells with the viral construct resulted in the cell line PDAC-2/Gal-4 and the control cell line (PDAC-2/mock) respectively. Moreover, in order to reduce the expression of Gal-4 we used RNA-mediated interference, as described previously [14]. Transfections were performed according to Invitrogen guidelines for reverse transfection in a 24-wells plate using 1 μl Lipofectamine RNAiMax and 100 μl Opti-MEM medium. A negative control (scramble-A together with negative control siRNA#1) was included in the experiments. Gal-4 mRNA and protein levels were measured using qRT-PCR and Western blotting at several time points during these experiments.

#### Migration and invasion assays

The effects of Gal-4 on migration and invasion were evaluated as described previously [40]. Migration was investigated using the LeicaDMI300B (Leica) migration station integrated with the Scratch-Assay 6.1 software (Digital-Cell Imaging Labs, Keerbergen, Belgium). Cells were plated at a density of 6×10^4^ cells/well onto 96-well plates, and after 24 hours, artificial wound tracks were created by scraping with a specific scratcher within the confluent monolayers. After removal of the detached cells by gently washing with PBS, the cells were treated with fresh medium. The ability of the cells to migrate into the wound area was determined by comparing the pixels in the images taken at the beginning of the exposure (time 0), with those taken after 4, 6, 8, 24 and 48 hours. In the migration studies using the Wnt inhibitor ICG-001, this inhibitor was added at IC50 concentration values in the refreshing medium.

For invasion assays, transwell chambers with polycarbonate membranes and 8 μm pores were used. The assays were performed through coated transwell filters, with 100 μL of 0.1 mg/mL collagen I solution. Hundred thousand cells were plated on the upper side of the filter. After 24 hours, cells that were migrated into the lower side were fixed with paraformaldehyde and stained with Giemsa in 20% methanol. The filters were photographed and cells were counted.

#### Inhibition of Wnt signaling studies

The effect of the Wnt inhibitor ICG-001 on cell growth was evaluated in PDAC-1, PDAC-1 transfected with Gal-4 siRNA (PDAC-1-Gal-4-siRNA), PDAC-2 and PDAC-2 transduced with the Gal-4 gene (PDAC-2-Gal-4), using the sulforhodamine-B (SRB) assay. The cells were plated in triplicate at a density of 5×10^3^ cells in a 96-well plate. After 24 hours, the cells were treated for 72 hours with ICG-001 (0.05-100 μM). Then plates were processed for the SRB assay, as described previously [41]. Optical density was measured at 540 nm using the Tecan SpectraFluor (Tecan, San Diego, CA). The 50% inhibitory concentration of cell growth (IC50) values of the drug was expressed as the concentration needed for a 50% reduction of cell growth after treatment relative to untreated controls, and calculated by non-linear least squares curve fitting (GraphPad PRISM, Intuitive Software for Science, San Diego, CA, USA).

#### Immunofluorescence of β-catenin

Immunofluorescence analysis of β-catenin was performed in cells seeded in 8-well chamber slides (Lab-Tek-II), fixed in formaldehyde/PBS and incubated in a methanol/acetone solution for 15 minutes. Cells were incubated with anti-β-catenin (1:500 dilution), washed in PBS and then probed with AlexaFluor 594 antibodies (Life Technologies). Nuclei were counterstained with 0.1 mg/ml 4',6-diamidino-2-phenylindole (DAPI, Life Technologies), as described previously [36].

#### Immunoprecipitation

In order to reveal the inhibitory effect of ICG-001 on the β-catenin/CBP complex, immunoprecipitation (IP) was performed in PDAC-2 nuclear extract, as described previously [35]. The immunoprecipitated antibody-antigen complex was also subjected to a reversal of the cross-link, followed by Western blot analysis as described above using either the polyclonal anti-β-catenin (dilution 1:1000), or polyclonal anti-CBP (1:1000) as primary antibodies (Santa Cruz Biotechnology, Dallas, TX, USA).

#### Statistical analysis

The relationship between Gal-4 and clinical outcome was evaluated by stratifying the patients with respect to the median expression value. Disease-free survival (DFS) was defined as the time from the date of diagnosis to the date of first relapse, while overall survival (OS) was calculated from the date of diagnosis to the date of death or last follow-up, obtained from medical records. Kaplan-Meier and log-rank methods were used to compare DFS and OS curves, using SPSS-20 (IBM, Chicago, IL).

All experiments were performed in triplicate and repeated at least twice. Data were expressed as mean values ± SEM and analysed by Student's t-test or ANOVA followed by the Tukey's multiple comparison test. The level of significance was P<0.05.

#### Ethics

All the patients gave their informed consent to the sample collection and analysis, and the study has received approval from the Ethics committee of Pisa University Hospital as a follow-up study of the research protocol entitled “Pharmacogenetics of gemcitabin-related genes in pancreas cancer: correlation with clinical outcome and tolerability” [42]. Animal experiments were carried out according to a protocol approved by the by the local animal welfare committee (Animal Experimental licensing Committee, DEC) of the VU University medical center, Amsterdam, The Netherlands [21].

## SUPPLEMENTARY MATERIAL AND FIGURES


