# Supplementary material for: Galectin-4 expression is associated with reduced lymph node metastasis and modulation of Wnt/β-catenin signalling in pancreatic adenocarcinoma
Source: Oncotarget. 2014 Jun 13;5(14):5335–49. doi: 10.18632/oncotarget.2104 (PMC4170638; doi:10.18632/oncotarget.2104)
Supplement: Supplementary file 1 [file oncotarget-05-5335-s001.pdf]

Galectin-4 expression is associated with reduced lymph node metastasis and modulation of Wnt/β-catenin signalling in pancreatic adenocarcinoma

Supplementary Material

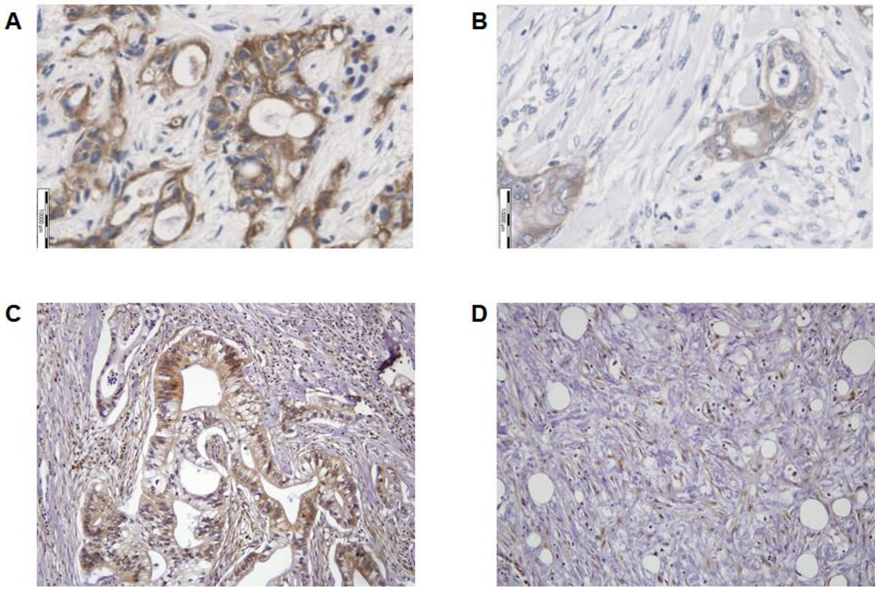

Supplementary Figure 1: Representative pictures of immunohistochemical analysis for Gal-4 expression in PDAC patients with low (A) and high (B) Gal-4 expression (original magnification, 40X), in PanIN (C, original magnification, 20X) and in the stroma tissue surrounding the tumor cells (D, original magnification, 20X)

A

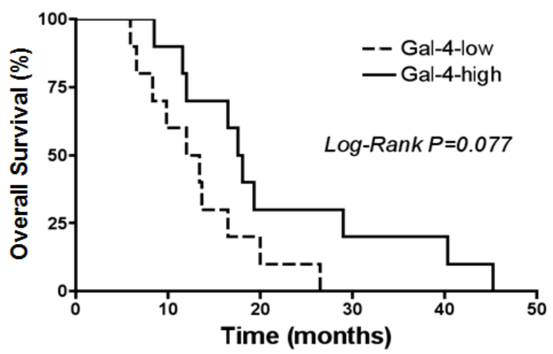

B

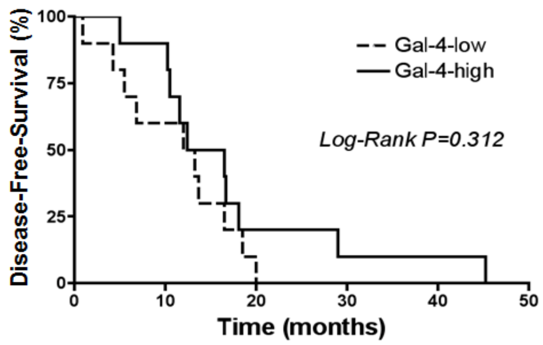

Supplementary Figure 2: Survival (A) and Disease-Free-Survival (B) Kaplan-Meier curves related to Gal-4 protein expression in the PDAC patients, grouped according to high/low vs median values. P values were calculated with two-sided log-rank test.
